# Diagnostic Contribution of SPECT/CT to Dual-Phase ^99m^Tc-MIBI Parathyroid Scintigraphy for Preoperative Localization in Primary Hyperparathyroidism

**DOI:** 10.3390/biomedicines14071627

**Published:** 2026-07-20

**Authors:** Vuslat Mumcu Çimen, Ebuzer Kalender

**Affiliations:** 1 Department of Nuclear Medicine, Gaziantep City Hospital, 27470 Gaziantep, Turkey; vuslatmumcu@hotmail.com; 2 Department of Nuclear Medicine, School of Medicine, Gaziantep University, 27310 Gaziantep, Turkey

**Keywords:** primary hyperparathyroidism, parathyroid scintigraphy, SPECT/CT, ^99m^Tc-MIBI, preoperative localization

## Abstract

**Background:** Accurate preoperative localization of hyperfunctioning parathyroid glands is essential for minimally invasive surgery in primary hyperparathyroidism (PHPT). Although dual-phase ^99m^Tc-MIBI planar scintigraphy is widely used, its diagnostic performance may be limited in small or ectopic lesions. Hybrid SPECT/CT imaging combines functional and anatomical information and may improve localization accuracy. The aim of this study is to evaluate the diagnostic contribution of SPECT/CT to dual-phase ^99m^Tc-MIBI planar scintigraphy for preoperative localization in patients with PHPT. **Methods**: This retrospective study included 128 patients with biochemically confirmed PHPT who underwent dual-phase ^99m^Tc-MIBI planar scintigraphy followed by delayed-phase SPECT/CT imaging before parathyroidectomy between January 2020 and May 2024. Imaging findings were compared with postoperative histopathological results. Sensitivity, specificity, positive predictive value (PPV), negative predictive value (NPV), and accuracy were calculated. The effects of lesion size, biochemical parameters, and ultrasonographic findings on imaging performance were also evaluated. **Results:** Histopathology confirmed parathyroid lesions in 122 of 128 patients (95.3%). The sensitivity, specificity, PPV, NPV, and accuracy of planar imaging were 57.4%, 66.7%, 97.2%, 7.1%, and 57.8%, respectively, whereas those of SPECT/CT were 92.6%, 66.7%, 98.3%, 30.8%, and 91.4%, respectively. SPECT/CT findings showed significant concordance with histopathology (*p* = 0.001), while planar imaging did not (*p* = 0.247). Among lesions smaller than 1 cm, sensitivities were 37.1% for planar imaging and 91.4% for SPECT/CT. SPECT/CT also provided superior anatomical localization, particularly in ectopic lesions and in patients with concomitant thyroid pathology. **Conclusions:** SPECT/CT demonstrated substantially higher diagnostic performance than dual-phase planar scintigraphy for preoperative localization in PHPT. Its incremental value was particularly evident in subcentimeter lesions, ectopic parathyroid adenomas, and patients with coexisting thyroid disease.

## 1. Introduction

Primary hyperparathyroidism (PHPT) is a clinical condition that develops due to uncontrolled parathyroid hormone (PTH) secretion as a result of the autonomization of the parathyroid glands. In terms of etiology, approximately 80–85% of cases are caused by a solitary parathyroid adenoma [1]. In PHPT cases with symptomatic hypercalcemia, surgery is still considered the first-choice definitive treatment [2]. Advances in imaging techniques and surgical capabilities have enabled the use of minimally invasive parathyroidectomy (MIP) in many centers [3]. In patients scheduled for surgery, preoperative localization studies should be performed to determine the location and number of hyperfunctioning parathyroid glands and to assess whether minimally invasive surgical treatment can be performed [4]. Preoperative localization studies can be conducted using invasive or non-invasive methods [5]. Technetium-^99m^–2-hexakis-methoxy-isobutyl-isonitrile (^99m^Tc-MIBI) scintigraphy, widely used in many nuclear medicine centers, is currently one of the most commonly utilized standard methods for imaging parathyroid lesions preoperatively, along with ultrasonography (US) [6]. Reported sensitivity rates for dual-phase ^99m^Tc-MIBI planar imaging range from 74% to 87% [7]. While this technique has both availability and advantages, it also has certain limitations. The aim of this clinical study is to investigate the contribution of single-photon emission computed tomography/computed tomography (SPECT/CT), a hybrid imaging technique that combines radionuclide imaging with X-ray-based computed tomography to provide both anatomical and functional information in a single image, to the diagnostic accuracy of dual-phase ^99m^Tc-MIBI planar scintigraphy in patients with a biochemical diagnosis of PHPT, using a larger patient cohort compared to previous studies in the literature.

Additionally, this study aims to compare the diagnostic performance of this modality with US findings and to evaluate the impact of lesion size and patients’ biochemical hormone levels on the diagnostic value of these imaging techniques.

## 2. Materials and Methods

**Ethical Approval:** This retrospective study was approved by the Clinical Research and Ethics Committee of Gaziantep University and conducted in accordance with the 1964 Declaration of Helsinki (Decision No: 2024/171, Date: 15 May 2024).

**Patient Selection:** This retrospective study included 128 patients with biochemically confirmed PHPT who underwent dual-phase ^99m^Tc-MIBI planar scintigraphy and SPECT/CT imaging during the preoperative period at the Nuclear Medicine Department of Gaziantep University. All patients had available histopathological data following parathyroidectomy.

**Imaging Protocol:** All imaging procedures were performed using a GE Healthcare Medical Systems NM/CT 860 dual-head gamma camera (General Electric, Milwaukee, WI, USA). Patients received an intravenous injection of 20 mCi (740 MBq) ^99m^Tc-MIBI, followed by planar imaging at 20 and 90 min. Anterior-positioned 10 min images were acquired from the level of the mandibular angle to the superior border of the heart, using a 128 × 128 matrix, a peak energy level of 140 keV with a 10% window, a zoom factor of 2.5, and a low-energy high-resolution (LEHR) parallel-hole collimator. Following the late-phase planar imaging, SPECT/CT imaging was performed using the same device. SPECT imaging was acquired using a LEHR collimator, a zoom factor of 1, a 140 keV peak energy level with a 10% window, and a 128 × 128 matrix in step-and-shoot mode with 6-degree steps, capturing 60 images over 360 degrees, with 30 s per image. Immediately after SPECT acquisition, CT imaging was performed using a low-dose CT system (4 slices) with X-ray tube settings of 120 kV and 10 mA. The scan was conducted in 3.75 mm standard slices with a helical pitch of 1 and full rotation mode. The scan time per slice was 6–7 s, and total CT acquisition was completed in approximately 4.5 min.

**Image Evaluation:** The obtained images were reviewed independently by two nuclear medicine physicians. In planar imaging, any focal uptake outside the physiological uptake regions between the mandibular angle and the superior border of the heart was considered positive for parathyroid pathology. In SPECT/CT images, these regions were examined in three dimensions, and any uptake corresponding to a lesion on CT, excluding physiological uptake areas, was recorded as positive. Findings were classified as negative or positive activity uptake, and for positive cases, the anatomical localization was documented. Postoperative pathology reports were reviewed through the hospital information and automation system. Histopathological diagnoses associated with PHPT (parathyroid adenoma, parathyroid hyperplasia, parathyroid carcinoma) were considered lesion-positive. Lesions diagnosed as normal parathyroid tissue or non-parathyroid tissue were considered lesion-negative. Imaging results were statistically analyzed by comparing them with pathology findings.

**Lesion Size Assessment:** Lesion size was determined using the maximum diameter (long axis) measured on preoperative neck US, which was routinely performed as part of the standard diagnostic work-up before surgery. Ultrasonographic measurements were used because they represent the lesion dimensions available at the time of preoperative imaging and clinical decision-making. Lesions were stratified into two groups (<1 cm and ≥1 cm) according to their maximum diameter. The 1 cm threshold was selected because subcentimeter parathyroid lesions are widely recognized as being more difficult to detect with conventional scintigraphic techniques, and previous studies have shown that SPECT/CT provides incremental diagnostic value particularly for lesions smaller than 1 cm [8,9]. This stratification was therefore intended to assess the incremental diagnostic value of SPECT/CT in detecting small parathyroid lesions. Although lesion volume may provide a more comprehensive assessment of lesion burden, volumetric measurements were not consistently available because of the retrospective design of the study and the lack of standardized three-dimensional ultrasonographic measurements across all patients. Therefore, maximum lesion diameter was selected as the most reproducible and consistently available parameter for lesion size assessment across the study cohort. Histopathological examination served as the reference standard for confirming the presence of parathyroid lesions. However, lesion size categorization was based on preoperative ultrasonographic measurements rather than pathological specimen dimensions because the primary objective of the study was to evaluate imaging performance under preoperative clinical conditions. Furthermore, pathological specimen dimensions may be influenced by surgical manipulation, tissue handling, and formalin fixation, resulting in discrepancies between in vivo imaging measurements and ex vivo pathological measurements.

**Statistical Method:** The normal distribution of numerical variables was tested using the Shapiro–Wilk test. Descriptive statistics were presented as mean ± standard deviation and median (minimum-maximum) for numerical variables and as frequency and percentage for categorical variables. Relationships between categorical variables were analyzed using the Chi-square test, while relationships between numerical variables were assessed using the Spearman rank correlation coefficient. Sensitivity, specificity, positive predictive value (PPV) and negative predictive value (NPV) and accuracy rates were calculated for diagnostic tests. All analyses were performed using SPSS version 22.0 for Windows, with *p* < 0.05 considered statistically significant.

## 3. Results

The study consisted of 128 patients diagnosed with PHPT who had not previously undergone neck surgery, including 102 (79.7%) females and 26 (20.3%) males. The patients’ ages ranged from 16 to 80 years, with a mean age (±SD) of 47.7 ± 13.2 years. Serum biochemical parameters measured within the 30 days before scintigraphic imaging were as follows: PTH levels ranged from 68.5 to 1376.1 pg/mL, with a mean of 265 (±192.9) pg/mL; calcium levels (Ca^+2^) ranged from 8.9 to 13.6 mg/dL, with a mean of 11.6 (±1.7) mg/dL; phosphorus (P) levels ranged from 1 to 7 mg/dL, with a mean of 2.4 (±0.7) mg/dL; and vitamin D levels ranged from 6.5 to 89.2 ng/mL, with a mean of 16.9 (±10.2) ng/mL (*normal ranges: PTH, 12–88* pg/mL*; Ca^2+^, 8.8–10.6* mg/dL*; P, 2.5–4.5* mg/dL). A single lesion was identified in all 128 patients undergoing parathyroidectomy. Histopathological analysis revealed parathyroid adenoma in 118 (92.2%) cases, parathyroid carcinoma in 4 (3.1%) cases, parathyroid hyperplasia in 1 (0.8%) case, normal parathyroid tissue in 3 (2.3%) cases, and non-parathyroid tissue in 2 (1.6%) cases. In patients diagnosed with parathyroid carcinoma, lesion sizes ranged from 15 to 45 mm (mean 29.3 ± 11.1 mm), and PTH levels ranged from 460.2 to 1376.1 pg/mL (mean 879 ± 397.3 pg/mL). Histopathology confirmed 95.3% of the lesions as positive, whereas planar imaging identified 56.2% and SPECT/CT identified 89.8% of the lesions (Figure 1, Table 1). Among the 9 (7%) lesions classified as ectopic, 1 was intrathyroidal, 6 were located in the upper-anterior mediastinum or related to the trachea, and 2 were in the paraesophageal area. SPECT/CT provided information about the location and anatomical relationships of all ectopic lesions, whereas planar imaging detected 7 of these 9 ectopic lesions but provided insufficient localization details.

**Evaluation Based on Biochemical Parameters:** In 72 patients with a lesion detected (+) by planar imaging, PTH levels ranged from 95.5 to 1376.1 pg/mL, with a mean of 288.1 (±228) pg/mL. In 56 patients with a lesion (−) on planar imaging, PTH levels ranged from 68.5 to 798.1 pg/mL, with a mean of 240 (±142.6) pg/mL. In 115 patients with a lesion (+) on SPECT/CT, PTH levels ranged from 92.7 to 1376.1 pg/mL, with a mean of 275.4 (±202.7) pg/mL. In 13 patients with a lesion (−) on SPECT/CT, PTH levels ranged from 68.5 to 396.7 pg/mL, with a mean of 192.8 (±89.9) pg/mL. When pathology results were compared with planar imaging findings, 70 cases were considered true positives (lesions detected by both pathology and planar imaging), 52 cases were false negatives (lesions detected by pathology but not by planar imaging), 2 cases were false positives (lesions detected by planar imaging but not by pathology), and 4 cases were true negatives (lesions not detected by either method). Chi-square analysis revealed no significant association between pathology findings and planar imaging results (*p* = 0.247). The sensitivity of planar imaging was 57.4%, specificity was 66.7%, PPV was 97.2%, NPV was 7.1%, and accuracy was 57.8%. When pathology results were compared with SPECT/CT findings, 113 cases were considered true positives, 9 were false negatives, 2 were false positives, and 4 were true negatives. Lesion locations identified as positive on SPECT/CT were consistent with surgical findings. Chi-square analysis showed a significant association between pathology and SPECT/CT results (*p* = 0.001). SPECT/CT had a sensitivity of 92.6%, specificity of 66.7%, PPV of 98.3%, NPV of 30.8%, and accuracy of 91.4%. Among the lesions, 70 (54.7%) were correctly identified as positive by both planar imaging and SPECT/CT, 9 (7%) were false negatives in both modalities, 1 (0.8%) was a false positive in both modalities, and 4 (3.1%) were true negatives in both modalities. Planar imaging missed 44 (34.4%) lesions that were detected by SPECT/CT. No lesion detected by planar imaging was missed by SPECT/CT (Table 2).

**Evaluation Based on Ultrasonographic Findings:** US data were available for 126 of the 128 patients. In 11 cases, no parathyroid lesion was identified on US; however, 5 of these lesions were detected by planar imaging, and 10 (including 3 ectopic lesions) were identified by SPECT/CT with precise anatomical localization. Additionally, in 2 cases, US detected a lesion that was not identified by either planar imaging or SPECT/CT. Among 13 patients with coexisting thyroid pathology (2 with nodular thyroid disease and 11 with multinodular goiter), histopathology confirmed parathyroid lesions. In these cases, 5 lesions were not detected by US (2 with nodular thyroid disease and 3 with multinodular goiter). Planar imaging detected only 2 of these lesions, whereas SPECT/CT identified all 5 lesions with precise anatomical localization, including 2 in ectopic locations.

**Evaluation Based on Lesion Size:** To assess the sensitivity of planar imaging and SPECT/CT concerning lesion size, 126 lesions with available US data were categorized into two groups based on their long axis: <1 cm and ≥1 cm. Lesion sizes ranged from 5 to 45 mm, with a mean of 13.6 (±6.3) mm. Among these, 91 (72.2%) lesions were ≥1 cm, and 35 (27.8%) were <1 cm.

Of the 35 lesions <1 cm, planar imaging detected 13 (37.1%) as positive and 22 (62.9%) as negative, whereas SPECT/CT detected 32 (91.4%) as positive and 3 (8.6%) as negative. The sensitivity of planar imaging for lesions <1 cm was 37.1%, whereas it was 91.4% for SPECT/CT. Among the 91 lesions ≥1 cm, planar imaging detected 59 (64.8%) as positive and 32 (35.2%) as negative, whereas SPECT/CT detected 83 (91.2%) as positive and 8 (8.8%) as negative. Chi-square analysis revealed a significant relationship between lesion size and planar imaging (*p* = 0.005), but no significant relationship was found between lesion size and SPECT/CT (*p* = 0.969).

## 4. Discussion

PHPT is a clinical condition resulting from the autonomous function of the parathyroid glands, leading to uncontrolled PTH secretion, which affects multiple organ systems and causes various symptoms, findings, and complications [1]. In a study by Ruda et al. [10] involving 20,225 PHPT patients, the etiology was found to consist of 88.9% single adenomas, 5.7% multiglandular hyperplasia, 4.1% double adenomas, and 0.7% parathyroid carcinoma. In our study, which included 128 PHPT cases, histopathological examination of lesions following surgery revealed 118 (92.2%) parathyroid adenomas, 4 (3.1%) parathyroid carcinomas, 1 (0.8%) parathyroid hyperplasia, 3 (2.3%) normal parathyroid tissues, and 2 (1.6%) non-parathyroid tissues. Surgical intervention is the primary treatment option for biochemically diagnosed and symptomatic hypercalcemic PHPT cases [2]. The fact that solitary adenoma is responsible for 80–85% of cases, along with advancements in imaging techniques and surgical procedures, has led to MIP becoming the preferred approach among surgeons [3]. Accordingly, preoperative localization studies should be performed to determine the number and location of hyperfunctioning parathyroid glands and assess the feasibility of minimally invasive surgical treatment [4]. Currently, ^99m^Tc-MIBI scintigraphy, in combination with US, is the most commonly used standard imaging modality in the preoperative period for identifying parathyroid lesions [6]. The sensitivity of US varies between 55% and 87%, depending on the location of the parathyroid lesion, with lower sensitivity in cases of ectopic parathyroid tissue or normocalcemic PHPT [11]. The sensitivity of dual-phase ^99m^Tc-MIBI imaging has been reported to range between 74% and 87% [7]. When used in conjunction with US, the sensitivity for detecting parathyroid lesions increases to 81–95% [12].

Recent advances in parathyroid imaging have demonstrated that newer modalities, particularly 18F-fluorocholine (^18^F-FCH) PET/CT and four-dimensional computed tomography (4D-CT), have demonstrated superior diagnostic performance than conventional scintigraphic techniques in selected clinical scenarios. Nevertheless, current evidence continues to support ultrasonography and ^99m^Tc-MIBI SPECT/CT as first-line localization modalities, while advanced imaging techniques are generally recommended for patients with negative or inconclusive conventional imaging findings or in more complex clinical settings. Therefore, evaluating the incremental diagnostic value of SPECT/CT over conventional planar scintigraphy remains clinically relevant, particularly for optimizing preoperative localization in routine clinical practice [9,13]. Accordingly, our study was designed to evaluate the additional diagnostic contribution of SPECT/CT over conventional dual-phase planar imaging in a real-world cohort of surgically confirmed PHPT patients. In our study, the sensitivity of dual-phase planar ^99m^Tc-MIBI imaging was found to be 57.4%. In a meta-analysis conducted by Ruda et al. [8], the sensitivity of dual-phase ^99m^Tc-MIBI scintigraphy for solitary adenoma was reported as 88.4%. However, in the same study, the sensitivity decreased to 44.5% and 30% in cases with four-gland hyperplasia or double adenomas, respectively. In our study, the sensitivity of dual-phase planar ^99m^Tc-MIBI imaging alone was found to be 57.4%, which is lower compared to similar studies in the literature.

The introduction of SPECT imaging enabled three-dimensional evaluation in parathyroid scintigraphy and improved the detection of ectopic, multiglandular, and small lesions compared with planar imaging [14]. However, despite better lesion localization, SPECT alone remains limited in providing precise anatomical details. By combining functional and anatomical imaging, SPECT/CT has been reported to improve lesion localization, particularly in ectopic adenomas, small lesions with low radiotracer uptake, and obese patients [8,15]. While the literature reports variable diagnostic performances for SPECT/CT compared with planar imaging [16], a meta-analysis by Wei et al. [17] demonstrated higher sensitivity and PPVs for SPECT/CT than for SPECT or planar scintigraphy and recommended its use before minimally invasive parathyroidectomy. Similarly, Pata et al. [18] reported that SPECT/CT improved lesion localization and reduced operative time compared with SPECT alone. In our study, SPECT/CT demonstrated markedly higher sensitivity and accuracy than planar imaging and showed stronger concordance with histopathological findings.

Ectopic parathyroid adenomas are observed in 9–22% of PHPT patients and are more common in persistent or recurrent disease [19]. Although the combined use of US, planar scintigraphy, and SPECT improves lesion detection [20], accurate anatomical localization may remain challenging. Öksüz et al. [21] demonstrated significantly higher lesion-based sensitivity for SPECT/CT than for planar scintigraphy, particularly in small adenomas and ectopic lesions. In our study, SPECT/CT successfully localized all ectopic lesions and provided detailed anatomical information, whereas planar imaging failed to adequately define lesion localization in several cases (Figure 2). Similarly, Serra et al. [22] reported that SPECT/CT contributed substantially to surgical planning, particularly in retrotracheal adenomas. These findings support the additional value of SPECT/CT in anatomically complex or ectopic lesions.

There is no consensus on the optimal timing for SPECT/CT and SPECT imaging. Both early- and delayed-phase imaging protocols have been used. However, due to the rapid washout observed in 15–40% of parathyroid adenomas, lesion detection becomes more challenging. To avoid missing lesions with early washout, early-phase SPECT imaging is often recommended in clinical settings [15,23]. Faster clearance is more common in hyperplastic glands [12]. In a study by Perez-Monte et al. [24], comparing early and late-phase SPECT images in 47 patients, early-phase images achieved a sensitivity of 91%, while the sensitivity dropped to 74% in the late-phase due to rapid washout. However, no significant difference was found between early and late-phase SPECT techniques in hyperplastic glands. In our clinic, considering radiation exposure, SPECT/CT imaging was only performed in the delayed phase, and the sensitivity for SPECT/CT was found to be 92.6%.

According to the literature, 8–33% of patients with PHPT have multiple gland hyperplasia (MGH). Hyperplastic glands are typically smaller than solitary parathyroid adenomas, which may reduce detection rates. In cases of MGH, the sensitivity of US and ^99m^Tc-MIBI is significantly lower compared to solitary adenomas. The use of SPECT/CT and 4D CT in these patients has been reported to provide more comprehensive information regarding localization and anatomical details, thereby increasing the likelihood of achieving a safe and successful operation [23,25]. Although the success rate of parathyroidectomy is over 95%, surgical failure remains one of the most common complications today [26]. Persistent hyperparathyroidism (pHPT) after parathyroidectomy occurs in 2–22% of cases and may recur in 1–10% of cases [27]. In patients presenting with perPHPT/recPHPT, scar formation and altered anatomy after previous surgeries pose significant technical challenges. The most common causes of perPHPT include MGH or missed ectopic parathyroid glands. Therefore, preoperative imaging is crucial in reoperative perPHPT/recPHPT cases [28,29]. Wimmer et al. [30] conducted a prospective study comparing ^99m^Tc-MIBI SPECT and ^99m^Tc-MIBI SPECT/CT imaging in 28 patients with HPT who had previously undergone neck surgery. The exact localization of abnormal glands was successfully predicted in 24 of 28 patients (86%) using SPECT/CT, whereas SPECT alone was successful in only 12 of 28 patients (43%, *p* < 0.004). SPECT/CT detected all three ectopically located pathological glands, while SPECT identified only one. Specificity was reported as 97% for SPECT/CT and 92% for SPECT, with an overall accuracy of 95% and 81%, respectively. Due to various factors in PHPT patients, the sensitivity of ^99m^Tc-MIBI scintigraphy has been reported to vary from 43% to 100%. Factors such as gland weight, preoperative serum PTH level, and plasma calcium levels are known to influence the sensitivity of ^99m^Tc-MIBI scintigraphy [31].

Ozkan et al. [8] reported higher lesion-based sensitivity and accuracy for SPECT/CT than for dual-phase planar scintigraphy and demonstrated that subcentimeter lesions missed on planar imaging could be successfully detected by SPECT/CT. Similarly, Bural et al. [32] showed that SPECT/CT provided superior localization performance for parathyroid adenomas smaller than 1 cm, mainly due to the additional anatomical information provided by CT imaging. In our study, 35 lesions had a long axis smaller than 1 cm, and SPECT/CT correctly detected 32 (91.4%) of these lesions, whereas planar imaging identified only 13 (37.1%). Furthermore, lesion size showed a significant association with planar imaging findings (*p* = 0.005) but not with SPECT/CT results (*p* = 0.969). These findings support the additional diagnostic value of SPECT/CT in the detection of subcentimeter parathyroid lesions (Figure 3).

Biochemical values and disease severity can affect the sensitivity of scintigraphy results. High serum calcium and PTH levels, as well as conditions such as vitamin D deficiency, increase the likelihood of positive scintigraphy results [15]. In a study by Ciappuccini et al. [33], in 94 PHPT patients who underwent dual-phase ^99m^Tc-MIBI planar scintigraphy followed by delayed-phase SPECT/CT, a significant relationship was found between high serum calcium and PTH levels, lesion size, and the likelihood of SPECT/CT being positive (*p* < 0.001). In our study, planar imaging demonstrated false-negative findings even in patients with elevated PTH levels, while SPECT/CT demonstrated superior ability to detect true positive cases even with lower PTH levels.

Concomitant thyroid disease is common in patients with PHPT and may reduce the diagnostic accuracy of both US and planar scintigraphy because of nonspecific ^99m^Tc-MIBI uptake in thyroid lesions [34,35]. In our study, SPECT/CT successfully detected all histopathologically confirmed lesions in patients with coexisting thyroid pathology, including ectopic lesions that were missed by US or planar imaging. Similarly, Shafiei et al. [36] reported higher sensitivity and accuracy for SPECT/CT than for SPECT or planar imaging in PHPT patients with nodular goiter. These findings suggest that SPECT/CT provides additional diagnostic value in patients with concomitant thyroid abnormalities.

## 5. Study Limitations

This study has several limitations. First, its retrospective single-center design may limit the generalizability of the findings. Second, the number of pathology-negative cases was relatively low, which may have affected the robustness of specificity and negative predictive value calculations. Third, only delayed-phase SPECT/CT imaging was performed; therefore, the potential additional contribution of early-phase SPECT/CT could not be evaluated. Finally, surgical outcomes such as operative duration or complication rates were not analyzed. In addition, a direct comparison with newer imaging modalities, such as ^18^F-FCH PET/CT or 4D-CT, was not possible because these techniques were not routinely available at our institution during the study period. Although recent studies have demonstrated promising diagnostic performance for these modalities, future prospective studies directly comparing them with dual-phase ^99m^Tc-MIBI SPECT/CT would help further define the optimal imaging strategy for patients with primary hyperparathyroidism [9,37].

## 6. Conclusions

SPECT/CT demonstrated substantially higher diagnostic performance than dual-phase planar scintigraphy for preoperative localization in patients with primary hyperparathyroidism. Its additional diagnostic value was particularly evident in ectopic lesions, subcentimeter parathyroid adenomas, and patients with concomitant thyroid pathology. These findings support the complementary role of SPECT/CT in improving lesion localization and preoperative evaluation in PHPT. Despite the emergence of newer imaging modalities, SPECT/CT continues to provide meaningful incremental diagnostic value over conventional planar scintigraphy and remains an important component of routine preoperative localization for patients with PHPT.

## Figures and Tables

**Figure 1:**
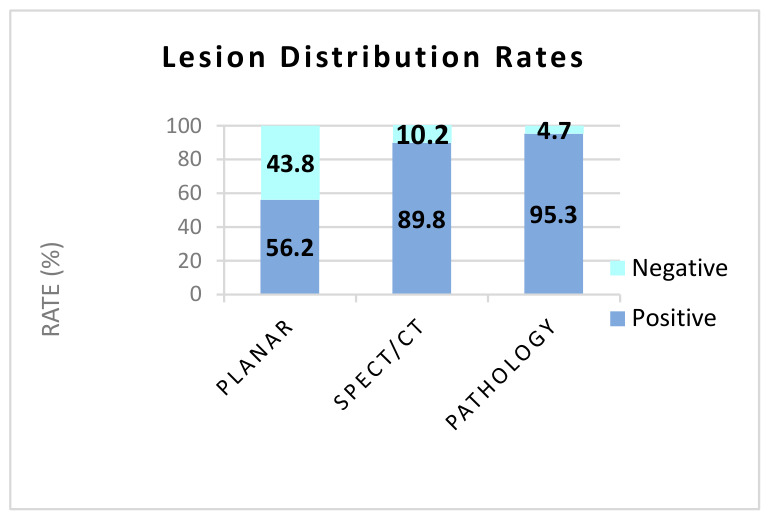
Distribution of Lesion Detection Rates by Imaging Method. SPECT/CT: single-photon emission computed tomography/computed tomography.

**Figure 2:**
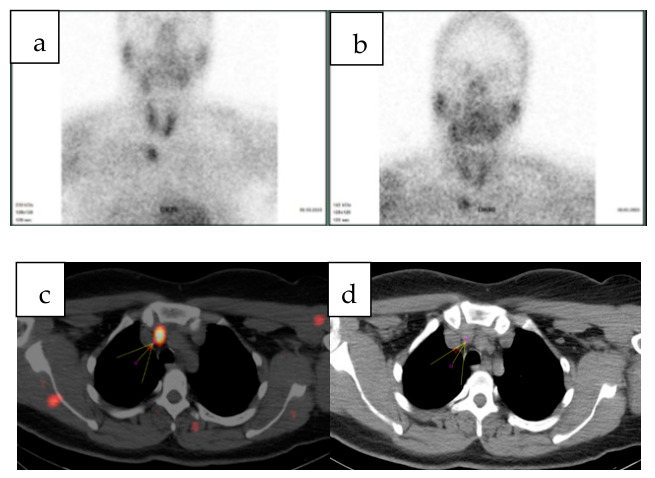
A 36-year-old female patient with biochemically confirmed primary hyperparathyroidism and negative ultrasonographic findings. Early planar imaging demonstrated focal ^99m^Tc-MIBI retention in the mediastinal region (**a**), which persisted on delayed images (**b**). Delayed-phase SPECT/CT revealed a focal uptake corresponding to a right anterior paratracheal lesion at the mediastinal inlet, consistent with a parathyroid adenoma (**c**,**d**). Histopathological examination confirmed parathyroid adenoma. SPECT/CT provided precise anatomical localization of the ectopic lesion.

**Figure 3:**
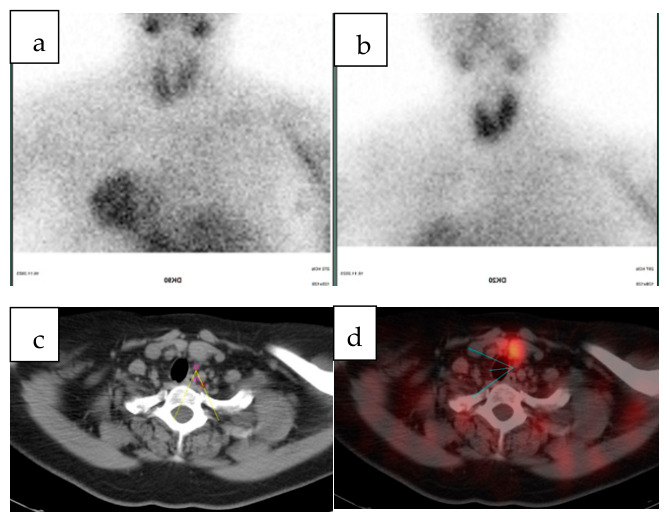
A 42-year-old female patient with primary hyperparathyroidism. Early and delayed dual-phase planar scintigraphy showed no distinct focal retention suspicious for parathyroid adenoma (**a**,**b**). Delayed-phase SPECT/CT demonstrated focal low-level ^99m^Tc-MIBI uptake corresponding to a nodular lesion located posterior to the left thyroid lobe (**c**,**d**). Histopathological evaluation confirmed parathyroid adenoma. SPECT/CT enabled the detection and anatomical localization of a subcentimeter lesion that was not clearly visualized on planar imaging.

**Table 1:** Distribution of Results According to Pathology and Imaging Methods.

	Lesion	n	%
**Planar Imaging**	**Positive**	72	56.2
	**Negative**	56	43.8
**SPECT/CT**	**Positive**	115	89.8
	**Negative**	13	10.2
Pathology	Positive	122	95.3
	Negative	6	4.7

n: number of lesions; SPECT/CT: single-photon emission computed tomography/computed tomography.

**Table 2:** Evaluation of the Results of Methods on a Lesion Basis.

	n	%
Planar (+); SPECT/CT (+); Pathology (+)	70	54.7
Planar (−); SPECT/CT (+); Pathology (+)	44	34.4
Planar (−); SPECT/CT (−); Pathology (+)	9	7.0
Planar (−); SPECT/CT (−); Pathology (−)	4	3.1
Planar (+); SPECT/CT (+); Pathology (−)	1	0.8

n: number of lesions; SPECT/CT: single-photon emission computed tomography/computed tomography.

## Data Availability

The data presented in this study are available on request from the corresponding author. The data are not publicly available due to institutional privacy regulations and the protection of patient confidentiality.
